# Near-Infrared Spectroscopy-Based Discriminant Analysis for the Classification of Coffee Quality in Dry Parchment and Green Coffee

**DOI:** 10.3390/molecules31091395

**Published:** 2026-04-23

**Authors:** Claudia Rocio Gómez, Aristófeles Ortiz, Valentina Osorio Pérez

**Affiliations:** National Center for Coffee Research, Cenicafé Km4 Chinchiná, Manizales 170009, Colombia; aristofeles.ortiz@cafedecolombia.com (A.O.); valentina.osorio@cafedecolombia.com (V.O.P.)

**Keywords:** sensory, absorbance, spectrum, profile, regression, discriminant, total lipids, caffeine

## Abstract

This study evaluates the potential of near-infrared spectroscopy (NIRS) combined with discriminant analysis to classify coffee quality based on sensory defects in dry parchment coffee (DPC) and green coffee. Spectral data were used to develop classification models, which were validated using both cross-validation and independent external datasets. Model performance was assessed using classification accuracy and Cohen’s kappa coefficient. The results demonstrate high classification accuracy for DPC (93.5%), with a Kappa coefficient indicating almost perfect agreement (κ = 0.90). In contrast, green coffee showed lower predictive performance (82.4%) and moderate agreement (κ = 0.55), reflecting the greater physicochemical complexity of this matrix. Importantly, the findings demonstrate that coffee quality can be reliably classified at the dry parchment stage, enabling early quality assessment without additional processing steps. This represents a significant advancement compared to previous studies, which have mainly focused on green or roasted coffee. Overall, these results highlight the potential of NIRS as a rapid, non-destructive, and objective tool for coffee quality assessment, with strong applicability in quality control and decision-making processes along the coffee production chain.

## 1. Introduction

Colombian coffee is widely recognized for its high quality, and this distinct sensory quality depends on multiple factors. The first factor is the composition of chemical compounds in coffee beans, which are transformed during roasting [1]. Other relevant factors include coffee variety, environmental conditions (such as climate and soil), and agronomic and postharvest practices, such as cultivation, crop management, and processing [2]. The sensory quality of coffee is evaluated through sensory analysis, which is carried out by trained judges according to specific attributes and standardized scoring protocols. Sensory analysis enables the identification of defective or off-flavor coffees, characterization of attributes, evaluation of intensity, and classification based on quality profiles [3,4]. Sensory defects are associated with inadequate practices, such as the collection of green beans, uncontrolled fermentation, drying interruptions or prolonged storage, which generate flavors such as immature, vinegary, musty, onion-like, earthy, vegetal, and papery (cardboard-like) [3,5,6,7].

Puerta [8] carried out a sensory quality analysis of samples from the Coffee Grower Cooperatives, over five months, which revealed the following sensory defects, in descending order: fermented (36%), woody (19%), immature (17%), and dirty-rough (14%), followed by aged and contaminated. Similarly, Osorio et al. [4] classified the main defects in Colombian coffee into four groups: fermented, rough, earthy and contaminated, which include the defects mentioned above [4,8]. Regarding the “aged” defect, Gallego et al. [5] performed a discriminant analysis and found that the influencing variables are the threshing yield factor (less than 71), stearic acid content (7.23–7.53%) and polyphenoloxidase (PFO) activity (0.000167–0.00019 U). Rendón et al. [9] also analyzed the “aged” defect and reported changes in the content of fatty acids (1.4–3.8 mg/g), lipid oxidation measured as TBARS (8.8–1.2 nmol MDA/g) and carbonyl groups (2.6–3.5 nmol/mg protein) [5].

Farah et al. [3] performed a correlation analysis between sensory quality and chemical composition. They reported that, for green coffee, trigonelline content decreased as the degree of sensory defects increased, with values ranging from 1.34 to 0.96 g/100 g (db). Another correlated compound class was total chlorogenic acids, which showed lower levels in sound green coffee (5.78 g/100 g db) and higher levels (7.02 g/100 g db) in defective samples. Pabón et al. [10] conducted a principal component analysis (PCA) and reported that earthy and fermented sensory defects are related to physical defects such as brocaded, black and vinegar beans. Regarding the chemical composition of green coffee, several authors have studied the relationships between chemical components and sensory attributes [11,12,13]. The lipid fraction of coffee, which includes total lipids and free fatty acids has been identified as an important determinant of sensory quality. Lipids and fatty acids act as carriers of aroma and flavor compounds and contribute to the body and texture of the beverage. In addition, specific fatty acids, such as linoleic acid and oleic acid, have been associated with sensory attributes such as aroma and acidity [14,15,16,17].

Alkaloids, particularly caffeine and trigonelline, represent an important class of chemicals compounds in coffee. These compounds are primarily associated with bitterness and, to a lesser extent, with other sensory attributes, such as aroma and acidity [18,19,20,21,22]. Sugars also play a key role in sensory quality, as they act as precursors of flavor, aroma, acidity and color. During roasting, sucrose is hydrolyzed into reducing sugars such as glucose and fructose, which contribute significantly to the taste and aroma of coffee [12,23,24]. Chlorogenic acids are considered precursors of the taste, acidity, astringency and bitterness in coffee. These compounds are naturally present in coffee beans, and the concentration of chlorogenic acids varies depending on coffee variety and roasting conditions [25,26,27].

Near-infrared spectroscopy (NIRS) is an analytical tool widely used for the evaluation, quality control and determination of chemical properties in agricultural products [28]. In coffee, NIRS has been used to differentiate varieties, roasting degrees, adulteration types, and both physical and sensory attributes as well as to quantify major chemical compounds; these applications have been developed for green coffee and roasted coffee [7,28,29,30,31,32,33,34,35,36]. The NIRS technique has evolved significantly in recent decades and is considered a precise and reproducible analytical method for both qualitative and quantitative analysis in the pharmaceutical, agri-food and chemical industries [37,38]. Its main advantages include rapid analysis, minimal or no sample preparation, non-destructive measurement, environmental friendliness, and the absence of chemical reagents [39,40].

To achieve the objectives of this research, sample data were analyzed using discriminant statistical analysis, evaluating the relationship between absorbance at each wavelength and sensory quality. The results were used to determine the linear combination of independent variables that maximized the separation between predefined classes [41,42]. In this type of calibration model, a confusion matrix is generated, allowing for the evaluation of classification performance. Correctly classified samples are referred to as true positives, whereas incorrectly classified samples are considered false positives. These results are used to calculate the percentage of success of the model [34,43,44].

This study presents calibration models developed to identify samples with and without sensory defects in both dry parchment coffee and green coffee. Additionally, the chemical composition of two evaluated coffee matrices (WSD and NSD) was analyzed.

Despite the increasing application of NIRS in coffee analysis, most studies have focused on single matrices, such as green or roasted coffee, and have typically addressed either chemical composition or quality classification independently. Moreover, limited studies have explored the integration of chemical and sensory information within a unified modeling framework. In addition, few studies have evaluated the performance of discriminant models across different coffee matrices under comparable conditions or explored alternative approaches such as RMS X residual-based model selection.

In this context, the present study aims to develop and evaluate NIRS-based discriminant models for the classification of coffee quality in both dry parchment coffee (DPC) and green coffee. The study also investigates the relationship between chemical composition and sensory defects, providing a more comprehensive and robust approach for coffee quality assessment under practical conditions.

## 2. Materials and Methods

The materials and methods selected for this research were designed to ensure an objective, reproducible, and consistent evaluation of coffee quality based on sensory defects and their correlation with the spectral characteristics. In this study, groups of coffee samples were initially established based on differences in quality and the most prevalent sensory defects at the country level. Subsequently, the samples were collected and subjected to sensory analysis by internationally trained and certified panels to ensure the reliability of the reference samples. The analysis was then carried out using near-infrared spectroscopy (NIRS), obtaining the spectral fingerprint of each sample. To develop the classification models, a principal component analysis (PCA) was first applied to explore the data structure and determine the potential separation between groups. Once the classification trends associated with quality were identified based on the spectral fingerprints, multiple discriminant models were evaluated, and the one with the best performance was selected. Finally, the performance of the selected model was evaluated, including cross-validation and independent external validation. Additionally, to complement the interpretation of the differences observed in the spectral fingerprints.

### 2.1. Coffee Samples

A total of 2054 dry parchment coffee (DPC) and 3834 green coffee samples were collected over a 23-month period, providing a comprehensive representation of Colombia’s diverse coffee-growing regions. The sampling frame encompassed 16 departments: Antioquia, Boyacá, Caldas, Cauca, César, Cundinamarca, Huila, Meta, Nariño, Norte de Santander, Quindío, Risaralda, Santander, Tolima, and Valle del Cauca. Samples were systematically characterized based on their sensory attributes, focusing on defect identification and quality profiling. Based on the sensory evaluation, samples were categorized into two primary groups:

With Sensory Defects (WSD): This group comprised four subgroups, over-fermented, rough, earthy, and contaminated, representing the most prevalent sensory defects in Colombian coffee.

No Sensory Defects (NSD): Samples in this group were classified into three distinct quality profiles (Table 1). Two coffee matrices, DPC and green coffee, were analyzed by NIRS.

### 2.2. Sensory Analysis

The sensory analysis of the samples was carried out by Q-Grader tasters certified by the Coffee Quality Institute (CQI), following a standardized laboratory protocol and the quality parameters established by the Specialty Coffee Association (SCA) [45]. The samples were carefully prepared prior to evaluation through threshing, selection of defect-free green beans, controlled roasting under standardized conditions, and subsequent cupping analysis. All sensory evaluations were conducted under controlled conditions to ensure the consistency and reproducibility of the results. The panel consisted of certified Q-Grader tasters with proven experience in coffee quality evaluation, ensuring high reliability of the sensory reference data. In addition, all tasting sessions adhered to standardized protocols for fragrance/aroma, flavor, aftertaste, acidity, body, balance, and defect identification. All participants voluntarily enrolled in the study after providing their written informed consent, in accordance with the ethical standards established by the institution.

### 2.3. Near-Infrared Spectroscopy (NIRS) Analysis

The samples were analyzed with NIRS XDS RCA (2012) equipment (FOSS, Hillerød, Denmark) in the wavelength range of 400–2498 nm; 110 g of DPC and green coffee were weighed into a rectangular reflectance cell 16 cm long, 5.0 cm wide and 5.0 cm high, with a quartz window. The samples were analyzed in duplicate, using the ISIscan program integrated with the analytical instruments. The number of samples used for model development and validation for each coffee matrix is presented in Table 2. This distribution reflects the natural composition of the dataset, including both samples with sensory defects (WSD) and without sensory defects (NSD), as well as the defined quality profiles. The relatively large number of samples per class strengthens model robustness and enables capturing the inherent variability of coffee under real production conditions.

### 2.4. Model Development

For spectral analysis and calibration model development, WinISI software (version 4, Infrasoft International, State College, PA, USA) was used. Spectral acquisition was performed using 32 scans per sample, and the resulting spectra were averaged to improve the signal-to-noise ratio. Background correction was carried out automatically by the instrument using a reference standard prior to spectral acquisition.

Prior to model development, principal component analysis (PCA) was performed to explore the structure of the spectral data and identify potential outliers. PCA is widely applied in NIR spectroscopy to reduce data dimensionality and detect anomalous samples based on spectral variability [28,40]. To improve spectral quality and reduce non informative variability, preprocessing techniques, including standard normal variate (SNV) and detrending (DT), were implemented. These methods correct scattering effects and baseline shifts in heterogeneous agricultural matrices such as coffee [46,47,48]. Outliers were initially identified using Mahalanobis distance (global H, GH > 3.0), a well-established criterion in NIRS to detect samples that deviate significantly from the population [49]. These samples were further evaluated using spectral residuals and leverage values to ensure robust detection and were subsequently excluded from the calibration dataset. Different mathematical treatments, including derivatives, wavelength selection, and smoothing, were evaluated. Derivative preprocessing enhances spectral resolution and helps remove baseline effects, whereas smoothing reduces high-frequency noise [49]. Additionally, different combinations of spectral pre-treatments and mathematical configurations available in the WinISI software were evaluated, including variations in derivatives, smoothing, and wavelength selection. Model performance was compared using several indicators provided by the software, such as PLS2 (%), correlation coefficient (R), maximum distance, Mahalanobis distance (GH), maximum spectral residual, and RMS X residual [50]. The final combination of treatments was selected based on its contribution to model stability, classification performance, and consistency between calibration and validation results [51]. After outlier removal, samples were randomly assigned to calibration and validation sets without enforcing a fixed proportion, in order to preserve the natural variability of the dataset. This strategy reflects real-world conditions, where class distributions are not necessarily balanced, and contributes to the robustness of the models.

Model selection was based on the consistency between calibration and validation results, prioritizing models that showed similar performance in both datasets. This approach reduces the risk of overfitting and ensures robust predictive capability under independent conditions [49]. Model performance was optimized by maximizing classification accuracy for each class (WSD and NSD) and for the defined quality profiles [50,52]. Performance was internally evaluated using cross-validation during model development and further assessed using an independent subset of samples reserved for validation [51]. Sensitivity and specificity were also calculated from the confusion matrix to evaluate the model’s ability to correctly identify samples with and without sensory defects. Sensitivity represents the proportion of correctly classified positive samples, whereas specificity reflects the proportion of correctly classified negative samples [53,54]. These complementary metrics provide a more comprehensive evaluation of classification performance, particularly in datasets with class imbalance, which is common in agricultural applications [55,56,57,58].

### 2.5. Model Performance Evaluation

Model performance was evaluated using overall accuracy and confusion matrix analysis. The confusion matrix provides a detailed representation of classification results by comparing reference (observed) and predicted classes, allowing the identification of correctly classified samples (true positives) and misclassified samples (false positives) [43,44].

In addition, Cohen’s Kappa coefficient (κ) was calculated to assess the agreement between predicted and reference classifications while accounting for agreement occurring by chance [59,60]. The coefficient was calculated as follows:

κ = (Po − Pe)/(1 − Pe)

where Po represents the observed agreement (overall accuracy), and Pe corresponds to the expected agreement by chance, calculated from the marginal totals of the confusion matrix. The interpretation of Kappa values followed the criteria proposed by Landis and Koch, where κ < 0 indicates poor agreement, 0.00–0.20 slight, 0.21–0.40 fair, 0.41–0.60 moderate, 0.61–0.80 substantial, and 0.81–1.00 almost perfect agreement.

### 2.6. External Validation of NIRS Models

External validation was performed using an independent set of samples that were not included in the calibration process. These samples were reserved prior to model development and used exclusively to evaluate model performance. The samples used in this study consisted of 69 defective and 55 non-defective dry parchment coffee samples, as well as 79 defective and 49 non-defective green coffee samples.

### 2.7. Prediction of Chemical Compounds by NIRS

Once the spectral information of the samples was obtained, the data were analyzed using calibration models to predict 10 chemical compounds in green coffee, using WinISI software (version 4, Foss Infrasoft International, USA). The performance of the quantitative models was evaluated using the relative prediction error, defined as:

Relative error = |1 − (X^−^_NIR/X^−^_ref)|

where X^−^_NIR represents the mean value predicted by NIRS and X^−^_ref corresponds to the mean value obtained using the reference laboratory method. This parameter is a dimensionless ratio that expresses the relative deviation between predicted and reference values. Values close to zero indicate higher model accuracy, whereas higher values indicate lower predictive performance. The results obtained for each compound are presented in Table 3 [33].

## 3. Results and Discussion

### 3.1. Quality of the Coffee Samples

**Figure 1:**
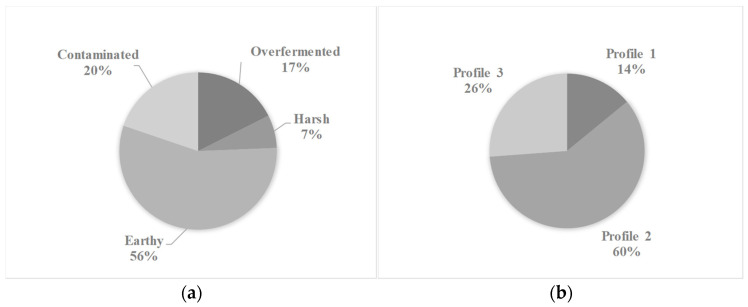
(**a**) Proportions of defect classes and (**b**) quality profile types for dry parchment coffee (DPC).

**Figure 2:**
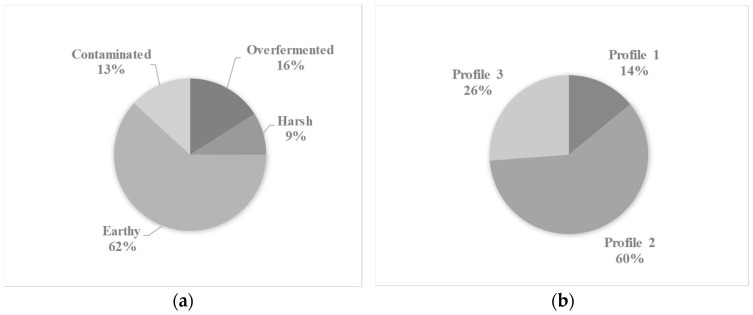
(**a**) Proportions of defect classes and (**b**) quality profiles in green coffee.

The analyzed samples originated from 16 departments of Colombia: Antioquia, Boyacá, Caldas, Cauca, César, Cundinamarca, Huila, Meta, Nariño, Norte de Santander, Quindío, Risaralda, Santander, Tolima and Valle del Cauca. The samples were analyzed over 23 months. The DPC and green coffee samples were characterized by defects and quality. Descriptive statistical analysis revealed that for both coffee matrices, the most common defect class was earthy (Figure 1a and Figure 2a), and the most common sensory profile was Profile 2 (Figure 1b and Figure 2b). The most common defects identified such as over-fermentation and earthy aromas are primarily associated with processing issues post-harvest, as well as deficiencies in infrastructure and process control at the farm level. These factors can lead to inappropriate fermentation conditions, deficient drying practices, and contamination, which ultimately affect the sensory quality of the coffee.

### 3.2. Spectral Analysis

**Figure 3:**
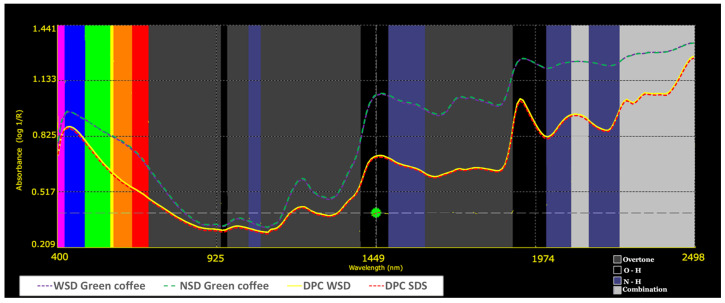
Mean near-infrared absorbance spectra of dry parchment coffee (DPC) and green coffee samples (400–2498 nm). Sample groups are identified by color as indicated in the legend. The multicolored band (400–700 nm) represents the visible region, while the remaining regions correspond to near-infrared absorptions (e.g., O–H, C–H, N–H).

Figure 3 shows the mean absorbance spectra for dry parchment coffee (DPC) and green coffee samples. Green coffee (represented by purple and green lines) exhibited higher absorbance values across the entire wavelength range compared to DPC (represented by yellow and red lines). The main absorption peak for green coffee was observed at approximately 1940 nm, whereas for DPC it was located around 1920 nm. These differences are primarily associated with variations in moisture content between matrices. The spectral region around 1900–1950 nm corresponds to O–H stretching vibrations, which are strongly influenced by water content [39,40]. Green coffee typically retains higher moisture levels than DPC, which explains the increased absorbance in this region.

In addition to water-related absorptions, differences in chemical composition—particularly lipids and carbohydrates—also contribute to spectral variability through C–H and O–H vibrational overtones [28,40]. Structural differences, such as the presence of the parchment layer in DPC, may further influence light scattering effects, affecting both absorbance intensity and peak position. These spectral differences are relevant for model development, as they provide the underlying variability required for the discrimination between samples with and without sensory defects. Variations associated with moisture and lipid-related absorption bands have been previously linked to compositional and quality differences in coffee and other agricultural matrices [28,40].

**Figure 4:**
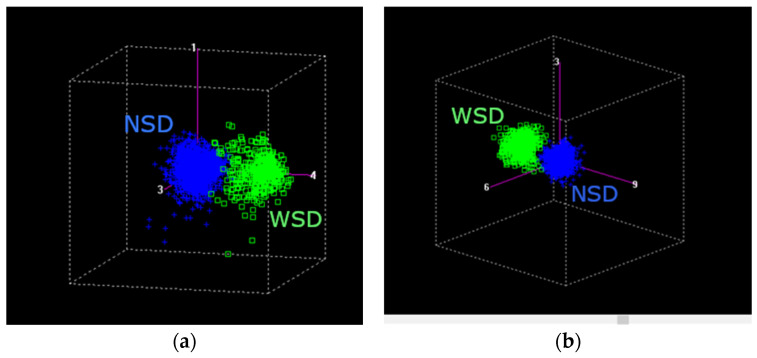
Principal component analysis (PCA) of (**a**) dry parchment coffee (DPC) and (**b**) green coffee samples, showing the distribution of samples in the principal component space. The axes correspond to selected principal components (e.g., PC1, PC3, and PC4 in (**a**), and PC3, PC6, and PC9 in (**b**)), chosen to enhance the visualization of sample separation. Green markers correspond to WSD samples, while blue markers represent NSD samples.

To further explore the internal variability of the dataset, PCA was applied separately to samples with sensory defects. PCA of the spectral data for dry parchment coffee (DPC) and green coffee samples identified 24 anomalous samples out of 2098 for DPC (Figure 4a) and 32 out of 4622 for green coffee (Figure 4b), which were excluded from further analysis.

The score plots (Figure 4) show a clear separation between samples with (WSD) and without sensory defects (NSD) for both matrices. The first two principal components explained 95% of the variance for DPC and 80% for green coffee, indicating that most of the relevant spectral information is captured within a reduced dimensional space.

The variability described by PC1 is mainly associated with compositional differences influencing NIR absorption, particularly those related to water and organic constituents such as lipids and carbohydrates [28,40]. The lower variance explained in green coffee reflects a more heterogeneous matrix, likely due to differences in composition and structural characteristics among samples.

**Figure 5:**
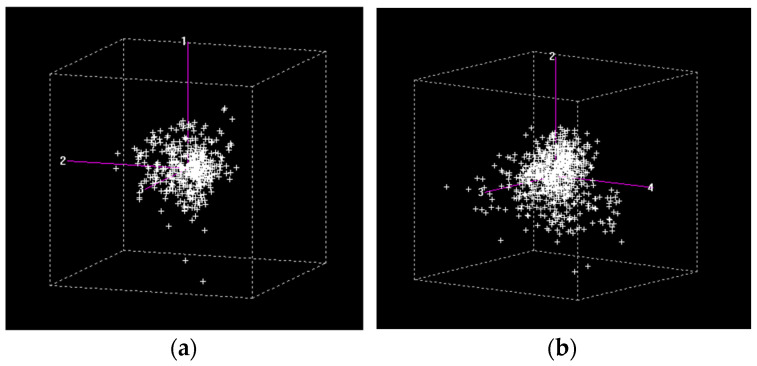
Principal component analysis (PCA) results for samples with sensory defects: (**a**) dry parchment coffee (DPC) and (**b**) green coffee. The distribution of samples is shown in the principal component space. The axes correspond to selected principal components (PC1, PC2, and PC3 in (**a**), and PC2, PC3, and PC4 in (**b**)), chosen to enhance the visualization of sample patterns.

In contrast, PCA applied to samples with sensory defects (WSD) did not reveal a clear internal structure (Figure 5). The absence of clustering suggests that defective samples do not share a uniform spectral pattern, which is consistent with the diverse physicochemical origins of sensory defects. Processes such as lipid oxidation, fermentation, and degradation of carbohydrates may generate overlapping spectral responses in the NIR region [28,40]. This behavior indicates that unsupervised methods such as PCA are limited for differentiating variability within defective samples, although they remain useful for initial data exploration.

**Figure 6:**
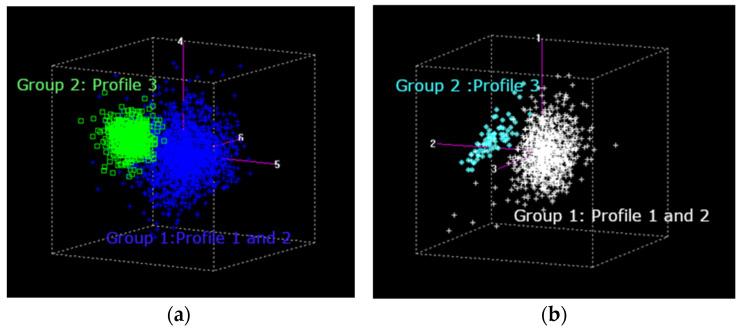
PCA results for samples without sensory defects: (**a**) DPC and (**b**) green coffee.

For samples without sensory defects (NSD), a different pattern was observed (Figure 6). In both DPC and green coffee, samples showed a tendency to separate into two groups: Group 1 (Profiles 1 and 2) and Group 2 (Profile 3). This separation reflects underlying differences in compositional and quality attributes within the NSD group. Profile 3 forms a more distinct cluster, suggesting a more consistent spectral signature compared to Profiles 1 and 2. Taken together, these results indicate that NIR spectroscopy can capture not only the presence or absence of sensory defects, but also variability within non-defective samples, supporting its application for classification into different quality profiles. Coffees with high sensory profiles are associated with higher sugar concentrations, better preservation of lipid composition, and alkaloids key precursors in the thermal reactions that occur during roasting. These compounds play a fundamental role in Maillard reactions, caramelization, and the generation of volatile aromatic compounds, which directly influence flavor complexity, aroma intensity, and mouthfeel. Consequently, the chemical composition of the green coffee matrix is a determining factor in its potential to express high sensory quality after roasting.

### 3.3. Development of Models

For each evaluated matrix and for the groupings obtained from PCA derived from the spectral analysis of the samples, discriminant classification models were developed and their performance indicators were compared. This approach enabled model selection based on quantitative and comparative criteria. Table 4 summarizes the results of the different model combinations generated using WinISI software, including the evaluated spectral pre-treatments and performance indicators. These results provide a comparative framework for the selection of the optimal model.

Among the evaluated strategies, the model based on the RMS X residual discriminant approach showed the best overall performance, achieving higher classification rates and a clearer separation between classes. This method classifies samples based on the root mean square (RMS) of spectral residuals, minimizing the distance between unknown samples and predefined class models. Its suitability for this study is associated with its ability to handle complex matrices such as coffee, where differences in composition and structural characteristics among samples influence spectral variability [49]. Overall, these results indicate improved discriminant capability and highlight the robustness of the selected model in capturing such differences.

Finally, it is important to contextualize the results obtained with the RMS-based approach against conventional classification methods widely employed in spectral data analysis, such as PLS-DA (Partial Least Squares Discriminant Analysis) and SVM-DA (Support Vector Machine Discriminant Analysis), which have demonstrated high performance in the classification of complex spectral datasets [61,62]. These supervised methods require careful calibration, validation, and parameter optimization to achieve reliable performance [63,64]. In contrast, the RMS-based method leverages the global variability of the spectral signal without the need for supervised training. This simplifies the analytical workflow, reduces methodological complexity, and facilitates interpretation of the results. While RMS-based classification may not always achieve the peak predictive accuracy of fully optimized PLS-DA or SVM-DA models, it provides a rapid and robust alternative, making it particularly suitable for applications that require fast and straightforward sample discrimination.

In this context, the RMS-based method is not intended to replace traditional supervised models but rather to complement them as an effective exploratory tool for the initial differentiation of samples with compositional and structural differences.

### 3.4. Discriminant Classification of Sensory Quality in Dry Parchment Coffee (DPC) and Green Coffee

The model with the best performance (Table 4) was further evaluated through confusion matrices to assess its classification ability. For dry parchment coffee (DPC), the model showed a high level of accuracy, with 997 true positives and 2 false positives for the NSD class (99.8%), and 404 true positives and 59 false positives for the WSD class (87.3%). The lower accuracy observed in samples with sensory defects suggests greater complexity in the spectral response of this group. In DPC, this variability may be mainly associated with physical factors related to the parchment structure. The presence of this layer, primarily composed of cellulose and hemicellulose, generates scattering effects in NIR radiation that can alter the spectral signal and reduce the model’s sensitivity to detect subtle differences between classes, as widely reported in heterogeneous solid matrices [49].

Cross-validation showed consistent behavior, with an overall accuracy above 90% and classification values similar to those obtained during calibration. The Kappa coefficient (κ = 0.90) indicated almost perfect agreement, confirming the robustness and stability of the model across different data subsets [59]. These results represent a significant advancement, as most NIR spectroscopy studies have focused on green or roasted coffee, whereas its application to dry parchment coffee has been limited. The ability to classify quality at this stage reduces the need for subsequent processes such as hulling, roasting, or sensory analysis, representing an operational advantage in terms of time, cost, and sample handling.

For green coffee, the confusion matrix showed an overall accuracy of 82.4%, with 84.5% for defective samples and 80.2% for non-defective samples, indicating lower discriminant performance compared to DPC. This decrease in performance can be attributed to the higher physicochemical heterogeneity of green coffee, particularly in terms of moisture content, chemical composition, and cellular structure, which directly affect the interaction of NIR radiation with the sample. As described by Osborne et al. [28] and Workman and Weyer [40], in complex biological matrices, the overlap of absorption bands associated with O–H, C–H, and N–H bonds—related to water, lipids, and carbohydrates—hampers the differentiation of specific spectral signals, reducing model sensitivity.

Cross-validation showed an overall accuracy of 78.4%, consistent with calibration results but lower than that observed for DPC, confirming reduced model stability for this matrix. This behavior has been previously reported in NIRS studies on green coffee. For example, Tolessa et al. [52] developed PLS models for sensory attributes such as acidity, sweetness, and aroma, reporting variability in predictive performance depending on the evaluated attribute. Similarly, Ribeiro et al. [53] showed that model accuracy depends on both sample complexity and the specific attribute analyzed. The Kappa coefficient (κ = 0.55) indicated moderate agreement, reinforcing the influence of intrinsic heterogeneity of green coffee on model performance. Overall, these results suggest that, although NIR spectroscopy is a useful tool for early-stage classification, its discriminant capacity in green coffee is limited by the compositional and structural complexity of the matrix. Recent studies provide further insight into the limitations observed in green coffee. Although near-infrared spectroscopy has demonstrated the ability to predict sensory attributes, predictive performance has been shown to depend strongly on the specific attribute and the intrinsic variability of the samples, particularly in less processed matrices [65]. Furthermore, large-scale FT-NIR studies have highlighted that spectral variability in green coffee is strongly influenced by compositional heterogeneity, requiring advanced preprocessing strategies to enhance signal discrimination. This complexity, associated with overlapping absorption bands and structural variability, has been identified as a key factor limiting model performance, which is consistent with the reduced discriminant capacity observed in the present study [66].

### 3.5. Classification by Quality Profiles

As previously mentioned, discriminant classification models were developed for both dry parchment coffee (DPC) and green coffee based on sensory quality profiles. Based on principal component analysis (PCA), two groups were defined: Group 1 (quality profiles 1 and 2) and Group 2 (quality profile 3) (Figure 6). For DPC, the model achieved an overall accuracy of 91.5%, with outstanding performance for Group 1 (99.2%) and lower accuracy for Group 2 (83.7%). Cross-validation confirmed this trend, with success rates of 90.7% and 79.4%, respectively, demonstrating the ability of NIRS to differentiate quality profiles in this matrix. The Kappa coefficient (κ = 0.87) indicated almost perfect agreement. The lower classification performance observed in Group 2 may be associated with the influence of the parchment structure, which, as reported for heterogeneous solid matrices [49], introduces scattering effects that hinder the detection of subtle spectral differences.

In green coffee, the model achieved an overall success rate of 94.6%, with a global error of approximately 5%. Unlike DPC, Group 2 showed near-perfect accuracy (99.7%), while Group 1 showed lower performance (89.6%). This trend was consistent in cross-validation (96.0% and 92.2%, respectively). The Kappa coefficient (κ = 0.85) also indicated almost perfect agreement. The relatively lower performance for Group 1 suggests that higher-quality samples may exhibit greater chemical and sensory variability, making spectral discrimination more challenging, consistent with the compositional complexity of green coffee described by Osborne et al. [28] and Workman and Weyer [40].

These findings confirm the potential of NIR spectroscopy for classifying coffee according to sensory quality profiles. In this context, Tolessa et al. [52] demonstrated that PLS-based models can predict sensory attributes with variable performance depending on the attribute evaluated, while Baqueta et al. [54] reported that prediction accuracy varies across individual sensory attributes. Together, these studies support the findings of the present work and reinforce the applicability of NIRS for objective coffee quality assessment. Recent literature reinforces the capability of near-infrared spectroscopy combined with multivariate analysis to model complex relationships between spectral signatures and sensory perception in coffee, supporting its application as a rapid and non-destructive tool for quality evaluation [65]. Recent studies have also demonstrated the potential of NIR spectroscopy combined with chemometric modeling for classification tasks related to post-harvest processing and coffee traceability. These approaches highlight the applicability of spectroscopic techniques not only for quality assessment but also for monitoring processing conditions along the value chain, reinforcing the broader applicability of NIRS-based models in the coffee sector [67]. These results highlight the potential of NIRS-based approaches not only for classification purposes but also for supporting decision-making processes in coffee quality control systems, particularly at early stages of the production chain.

### 3.6. External Validation

To evaluate the robustness of the model beyond calibration data, an external validation was performed using an independent set of samples not included in model development. For dry parchment coffee, 69 defective and 55 non-defective samples were used, achieving an overall accuracy of 91.1%. For green coffee, 79 defective and 49 non-defective samples were evaluated, obtaining an overall accuracy of 92.2%. The results were consistent with those obtained during calibration and cross-validation, confirming the stability, generalization capacity, and practical applicability of the proposed models. Furthermore, the models did not exhibit overfitting and effectively captured the relevant variability of the analyzed matrices. The robustness observed in the external validation is consistent with recent developments in NIR-based modeling approaches. The use of independent validation sets has been emphasized as a critical step to ensure model generalization and avoid overfitting, particularly in spectroscopic applications involving complex biological matrices [65]. In addition, studies based on large and diverse spectral datasets have demonstrated that model robustness and transferability improve when variability is adequately represented during model development, supporting the reliability of the models obtained in this study [66].

### 3.7. Chemical Composition of NSD and WSD Green Coffee

The chemical composition of WSD and NSD samples was estimated by the NIRS-based prediction models [35]. The results were first subjected to descriptive statistical analysis to identify trends, and then, multiple comparisons of the means were performed via the Tukey test. The results revealed significant differences for the ten tested chemical compounds. Table 5 shows the results of the analysis. Next, the characteristics of the coffee classes evaluated by groups of chemical compounds are described. The chemical composition of coffee beans is directly associated with their quality, as key components such as sugars, lipids, proteins, and alkaloids act as precursors for the development of flavor and aroma during roasting. Variations in these compounds influence the formation of desirable volatile and non-volatile compounds through Maillard reactions, caramelization, and other thermal processes, which directly affect sensory attributes such as sweetness, acidity, balance, and final sensory quality. Therefore, the intrinsic chemical profile of the green coffee bean is a fundamental determinant of its potential to achieve a high-quality sensory profile.

#### 3.7.1. Alkaloids

The mean caffeine content in the NSD samples was slightly lower than that in the WSD samples, with average values of 1.10% and 1.09%, respectively. These values are within the range reported by other authors, such as Villegas et al. [36], who reported values ranging from 1.03% to 1.52% (db), and Hagos et al. [55], who reported values ranging from 0.96% to 1.23% (db). Regarding trigonelline, the NSD samples had a mean content of 0.80%, and the WSD samples had a content of 0.81% (wb). Similar results were reported by Farah et al. [3], with an average value of 0.96%, and Gallignani et al. [19], with a value of 1.0%.

#### 3.7.2. Sucrose

WSD coffee presented lower average sucrose contents than NSD coffee, with values of 7.39% and 7.55% (wb), respectively. These results are consistent with previous studies carried out on NSD green coffee. Barbosa et al. [56] reported a similar sucrose content in NSD green coffee, with a value of 8.2% (db). Furthermore, Knopp et al. [57] analyzed the sugar content in arabica coffee of different origins and detected higher relative contents of sucrose, with average values between 7.6% and 8.2% (db).

#### 3.7.3. Total Chlorogenic Acids

NSD coffee samples presented a higher content of total chlorogenic acids than WSD coffee samples; the average value obtained for the WSD group was 3.45%, and for NSD coffee, the average was 3.52% (wb). These results differ from those reported by Gallego et al. [5], who found no significant differences in the content of total chlorogenic acids between coffee with the “aged” defect and NSD coffee, with average values of 4.5% and 4.4%, respectively. However, the total CQA contents in NSD coffee are close to those reported by other researchers, such as Gómez et al. [33], who found an average CQA content of 4.2% (db), but lower than those reported by Villegas et al. [36], who found an average value of 5.23%.

#### 3.7.4. Total Lipids

The total lipid content was greater in the WSD coffee samples, with an average value of 12.0%, than in the NSD coffee, which presented an average value of 11.78% (wb). These results coincide with those reported by Gallego et al. [5], who also found significant differences in total lipid content between coffee samples with the “aged” defect and NSD samples, with values of 11.87% and 11.0% respectively. Similarly, Rendón et al. [9] stored coffee for 15 months and determined that the coffee developed an “aged” defect and that the total lipid content increased compared with the initial value of 12%, which established that the increase was due to oxidation.

#### 3.7.5. Free Fatty Acids

WSD coffee presented higher contents of arachidic and stearic fatty acids, with values of 4.04% and 8.70%, respectively, than NSD coffee, which showed values of 3.64% and 8.03% (wb). On the other hand, NSD coffee presented higher contents of oleic, palmitic and linoleic fatty acids, with average values of 9.64%, 43.42% and 35.99% (wb), respectively (Table 5). These results agree with those of Gallego et al. [5], who also identified significant differences in fatty acids in samples with the “aged” sensory defect. In addition, in this study, higher contents of linoleic and stearic fatty acids were detected, with values of 38.25% and 7.53% (db), respectively. Rendón et al. [9] also found a significant increase in fatty acids and related this change to the loss of sensory quality. The prediction accuracy of the NIRS models for each chemical component, expressed as relative prediction error, is presented in Table 3. These values correspond to the overall performance of the calibration models and are not specific to individual sample groups.

Although statistical differences were observed for several chemical compounds (Table 6), the magnitude of these differences was generally low. However, the higher variation observed for arachidic fatty acid suggests a potential link with the formation of sensory defects. Arachidic fatty acid is a long-chain saturated fatty acid associated with the lipid fraction of coffee. Changes in lipid composition can be related to oxidative processes and biochemical transformations occurring during postharvest handling and storage [5,9]. Lipid oxidation is known to generate volatile compounds that negatively affect sensory attributes such as aroma and flavor [9,14,15,16,17]. Additionally, microbial activity during uncontrolled fermentation processes may influence lipid metabolism, contributing to changes in fatty acid composition [5]. Therefore, the observed increase in arachidic fatty acid in WSD samples could be associated with degradation processes linked to the development of sensory defects. These results indicate that, although most chemical differences are small, specific compounds such as arachidic fatty acid may act as indicators of quality deterioration and contribute to the discrimination between coffee samples with and without sensory defects.

#### 3.7.6. Chemical Composition of NSD Green Coffee

The NSD green coffee samples presented one of three quality levels (Table 1): Profile 1, “extraordinary” coffee; Profile 2, special; and Profile 3, standard. The quality profiles presented significant differences (Table 7). Profile 1 and Profile 2 were statistically the same but different from Profile 3 in terms of total lipid content and oleic and stearic fatty acid contents. Profile 3 showed a higher total lipid content (12.0%) than Profile 1 and Profile 2. Osorio et al. [12] reported that the total lipid content in green coffee at different stages of maturity ranged from 9.50% to 11.8%, and Echeverri-Giraldo et al. [68] reported values between 10.53% and 12.81%; thus, the values determined in this research for NSD coffee are within the reference range. With respect to the sucrose content, Profile 1 was significantly different from Profiles 2 and 3; Profile 1 showed a lower average sucrose content (7.44%). The chemical compounds that significantly differed among the three profiles were total chlorogenic acids and arachidic and palmitic fatty acids. The contents for total chlorogenic acids ranged from 3.39% to 3.67%, those for arachidic fatty acids ranged from 3.52% to 3.78%, and those for palmitic fatty acids ranged from 43.09% to 43.63%. The values obtained in this research are within the ranges reported by Osorio et al. [12], who found chlorogenic acid contents between 3.57% and 5.10%, arachidic fatty acid contents between 3.77% and 5.85%, and palmitic fatty acid contents between 38.85% and 45.27%. The compounds that did not show differences in content across the three quality profiles were caffeine, trigonelline and linoleic fatty acid. Osorio et al. [11] obtained caffeine contents of 1.09% to 1.16%, trigonelline contents of 0.87% to 0.88% and linoleic fatty acid contents of 33.03% to 34.9%, confirming the present findings.

### 3.8. Chemical Composition of WSD Green Coffee

The WSD samples with sensory defect were made up of the presented four main groups of defects identified in the sensory analysis: Group a, overfermented; Group b, rough; Group c, earthy; and Group d, contaminated, as shown in Table 1. The chemical information according to group of sensory defects was analyzed with the Tukey test; the results are presented in Table 8. The compounds that presented significant differences between the identified groups of sensory defects were total lipids and oleic fatty acid, with Group 1 (overfermented) having a higher content (average 12.45%) than the other groups. The group with the highest oleic fatty acid content was Group 4 (contaminated). Table 8 lists the minimum and maximum values, mean, standard deviation and group or result of the Tukey statistical analysis according to chemical compound and group of defects.

#### Overall Interpretation

The results obtained in this study confirm that near-infrared spectroscopy (NIRS), combined with discriminant analysis, provides a robust framework for classifying coffee samples according to sensory defects and quality profiles. The developed models achieved high classification performance for both dry parchment coffee and green coffee, enabling reliable discrimination between samples with and without sensory defects. Although the differences in chemical composition between classes were generally small, certain compounds, particularly arachidic fatty acid, showed a clearer association with quality deterioration. This suggests that specific components of the lipid fraction may contribute to the differentiation of defective samples.

A notable strength of this work lies in the use of samples collected from multiple coffee-producing regions in Colombia. This diversity introduces variability in environmental and processing conditions, which strengthens the robustness of the models and supports their applicability under real production scenarios. An additional contribution is the successful classification of coffee quality at the dry parchment stage. This finding demonstrates that it is possible to identify sensory quality without requiring threshing, roasting, or cupping, which represents a significant advantage in terms of operational efficiency. Furthermore, the integration of dry parchment coffee and green coffee within the same modeling strategy provides a broader analytical scope compared to previous studies focused on a single matrix. This expands the potential of NIRS as a tool applicable across different stages of coffee processing.

## 4. Conclusions

Unlike most studies reported in the literature, which have focused on a single matrix and regression models for specific sensory attributes, this study proposes a discriminant classification approach applicable to both dry parchment coffee and green coffee. This approach enables quality evaluation at early stages of processing, particularly in dry parchment coffee, representing a relevant contribution from an operational perspective. The integration of both matrices within a single modeling framework highlights the versatility of NIR spectroscopy and expands its application potential along the coffee production chain. From a chemical standpoint, the observed classification capability is associated with differences in sample composition and structure, which affect the interaction of NIR radiation with O–H, C–H, and N–H bonds related to water, lipids, and carbohydrates.

Overall, the results position NIR spectroscopy as a robust, rapid, and non-destructive tool for objective coffee quality evaluation, with strong potential for implementation in production environments. Furthermore, the developed models show high potential for application in quality control systems at key points in the supply chain, such as collection centers or export ports. In this context, their integration into NIRS equipment would enable rapid and objective verification of Colombian coffee quality prior to export, contributing to product standardization and traceability.

## Figures and Tables

**Table 1:** Description of the samples analyzed by NIRS.

Matrix	Features:		
With sensory defects—WSD	Overfermented: Pulp, vinegar, fermented, musty, and onion		
	Rough: Immature, pungent and rough, and cereal-like		
	Earthy: Earthy, mold and aged		
	Contaminated: Phenol, smoke, contaminated and chemical		
No sensory defects—NSD	Profile 1	Extraordinary	>84 points
	Profile 2	Special	82–84 points
	Profile 3	Standard	79–82 points

**Table 2:** Number of samples per coffee matrix used for the prediction models.

Coffee Matrix	Model Type	Class/Group	Number of Samples
Dry parchment	WSD and NSD classification	WSD *	554
		NSD *	1500
	NSD quality classification	Profile 1 and 2	1725
		Profile 3	727
Green	WSD and NSD classification	WSD	829
		NSD	3005
	NSD quality classification	Profile 1 and 2	901
		Profile 3	447

* WSD: with sensory defects; NSD: no sensory defects.

**Table 3:** Relative prediction error by chemical compound.

Chemical Compound	Relative Prediction Error *
Total lipids	0.003
Caffeine	0.015
Trigonellin	0.027
Sucrose	0.007
Total chlorogenic acids (CQA)	0.007
Palmitic fatty acid	0.007
Linoleic fatty acid	0.005
Oleic fatty acid	0.001
Stearic fatty acid	0.008
Arachidic fatty acid	0.002

* Relative prediction error (dimensionless).

**Table 4:** Performance of discriminant classification models for different combinations of spectral pre-treatments and coffee matrices.

Coffee Matrix	Model Type	Mathematical Treatment *	Pls2 (%)	Correlation Coefficient (R) (%)	Maximum Distance (%)	Mahalanobis Distance (GH) (%)	Maximum Spectral Residual (%)	RMS X Residual (%)
DPC	WSD vs. NSD classification	3, 3, 3, 1	0	53.1	39.4	56.4	56.9	93.5
	Quality classification (Group 1 and 2)	4, 3, 3, 1	5.7	58.9	41.9	59.2	57	91.5
Green coffee	WSD vs. NSD classification	1, 4, 4, 1	45.8	68.8	44.4	51.9	70.1	82.4
	Quality classification (Group 1 and 2)	3, 4, 4, 1	45.5	62.9	47.3	56.8	69.4	94.1

* Mathematical treatment is expressed as: derivative order, gap, first smoothing, and second smoothing, according to WinISI software conventions.

**Table 5:** Chemical composition by type of green coffee estimated by the NIRS technique.

Chemical Compound	Class	Minimum (%)	Maximum (%)	Mean (%)	Standard Deviation	*p* Value
Caffeine	NSD	0.83	1.35	1.10 B	0.08	0.015
	WSD	0.65	1.54	1.09 A	0.11	
Trigonellin	NSD	0.66	0.95	0.80 B	0.05	0.001
	WSD	0.65	1.02	0.81 A	0.06	
Sucrose	NSD	6.56	8.70	7.55 B	0.39	0.0001
	WSD	6.10	8.52	7.39 A	0.43	
Total chlorogenic acids	NSD	2.84	4.36	3.52 B	0.37	0.0001
	WSD	2.53	4.51	3.45 A	0.36	
Total lipids	NSD	9.27	14.11	11.78 B	0.77	0.0001
	WSD	8.89	15.43	12.00 A	1.30	
Arachidic fatty acid	NSD	1.90	5.46	3.64 A	0.69	0.0001
	WSD	1.21	5.24	4.04 B	0.74	
Oleic fatty acid	NSD	8.42	10.88	9.64 B	0.41	0.0001
	WSD	7.84	10.69	9.26 A	0.49	
Stearic fatty acid	NSD	6.28	9.98	8.03 A	0.72	0.0001
	WSD	5.99	10.61	8.70 B	0.90	
Palmitic fatty acid	NSD	40.41	46.83	43.42 B	1.20	0.0001
	WSD	38.54	48.38	42.99 A	1.76	
Linoleic fatty acid	NSD	28.96	39.91	35.99 B	1.27	0.0001
	WSD	25.62	40.19	35.23 A	2.22	

*p* Values correspond to one-way ANOVA. Different letters indicate significant differences according to Tukey’s post hoc test (*p* < 0.05).

**Table 6:** Chemical compounds, average contents by coffee class and corresponding percentages (%).

Compound	WSD	NSD	10% Decimal Average (Difference)	Criterion
Total lipids (%)	12.00	11.78	1.19	0.22
Caffeine (%)	1.09	1.10	0.11	0.01
Trigonellin (%)	0.81	0.80	0.08	0.01
Sucrose (%)	7.39	7.55	0.75	0.16
Total chlorogenic acids (%)	3.45	3.52	0.35	0.08
Arachidic fatty acid (%)	4.04	3.64	0.38	0.40
Oleic fatty acid (%)	9.26	9.64	0.94	0.38
Stearic fatty acid (%)	8.70	8.03	0.84	0.67
Palmitic fatty acid (%)	42.99	43.42	4.32	0.43
Linoleic fatty acid (%)	35.23	35.99	3.56	0.76

**Table 7:** Chemical composition of coffee by quality profile.

Chemical Compound	Quality Profile	Minimum (%)	Maximum (%)	Mean (%)	Standard Deviation
Total lipids	1	9.27	14.11	11.74 A	0.80
	2	9.50	14.00	11.67 A	0.73
	3	9.65	14.05	12.04 B	0.75
Caffeine	1	0.93	1.31	1.11 A	0.08
	2	0.83	1.31	1.11 A	0.08
	3	0.88	1.35	1.08 A	0.09
Trigonellin	1	0.66	0.92	0.79 A	0.05
	2	0.66	0.94	0.80 A	0.05
	3	0.70	0.95	0.81 A	0.05
Sucrose	1	6.60	8.63	7.45 A	0.40
	2	6.76	8.70	7.56 B	0.39
	3	6.56	8.54	7.58 B	0.38
Total chlorogenic acids	1	2.87	4.18	3.39 A	0.33
	2	2.87	4.22	3.50 B	0.36
	3	2.84	4.36	3.67 C	0.39
Arachidic fatty acid	1	1.90	5.46	3.78 C	0.61
	2	2.02	5.23	3.64 B	0.67
	3	1.91	5.08	3.52 A	0.77
Oleic fatty acid	1	8.83	10.84	9.74 A	0.36
	2	8.48	10.88	9.68 A	0.42
	3	8.42	10.61	9.46 B	0.38
Stearic fatty acid	1	6.42	9.98	8.16 B	0.66
	2	6.28	9.57	8.00 B	0.68
	3	6.37	9.77	7.99 A	0.83
Palmitic fatty acid	1	40.73	46.32	43.09 A	1.07
	2	40.41	46.83	43.43 B	1.20
	3	40.57	46.27	43.63 C	1.23
Linoleic fatty acid	1	28.96	39.34	36.08 A	1.36
	2	30.56	39.63	35.96 A	1.23
	3	30.67	39.91	35.99 A	1.30

*p* Values correspond to one-way ANOVA. Different letters indicate significant differences according to Tukey’s post hoc test (*p* < 0.05).

**Table 8:** Chemical composition of WSD coffee by group of sensory defects.

Statistical	Group	Minimum (%)	Maximum (%)	Mean (%)	Standard Deviation (n − 1)
Total lipids	a	9.67	15.43	12.45 B	1.10
	b	9.30	15.09	12.09 AB	1.29
	c	8.96	15.26	11.89 A	1.32
	d	8.89	14.33	11.89 A	1.30
Caffeine	a	0.78	1.54	1.12 A	0.10
	b	0.65	1.49	1.11 A	0.18
	c	0.69	1.35	1.09 A	0.10
	d	0.70	1.47	1.06 A	0.12
Trigonellin	a	0.67	0.98	0.81 A	0.07
	b	0.69	0.97	0.82 A	0.07
	c	0.65	0.98	0.81 A	0.06
	d	0.74	1.02	0.83 A	0.05
Sucrose	a	6.58	8.37	7.31 A	0.36
	b	6.21	8.37	7.40 A	0.52
	c	6.10	8.52	7.42 A	0.42
	d	6.48	8.41	7.33 A	0.48
Total chlorogenic acids	a	2.53	4.27	3.39 A	0.33
	b	2.99	4.21	3.47 A	0.32
	c	2.90	4.51	3.46 A	0.37
	d	2.98	4.34	3.43 A	0.35
Arachidic	a	1.21	5.13	4.06 A	0.74
	b	2.74	5.16	4.22 A	0.63
	c	2.10	5.24	4.00 A	0.76
	d	1.90	5.12	4.05 A	0.75
Oleic fatty acid	a	7.84	9.89	9.08 A	0.44
	b	8.30	10.47	9.23 AB	0.50
	c	8.05	10.69	9.29 B	0.49
	d	8.56	10.40	9.39 B	0.48
Stearic fatty acid	a	5.99	10.40	8.67 A	0.86
	b	6.78	10.36	8.86 A	0.86
	c	6.55	10.61	8.65 A	0.90
	d	6.38	10.50	8.85 A	0.95
Palmitic fatty acid	a	40.10	48.04	43.12 A	1.96
	b	40.34	47.47	42.71 A	1.62
	c	39.45	48.18	43.07 A	1.77
	d	38.54	47.49	42.66 A	1.52
Linoleic fatty acid	a	26.65	39.75	35.39 A	2.24
	b	28.98	39.07	34.98 A	2.21
	c	25.62	40.19	35.18 A	2.24
	d	29.71	38.66	35.52 A	2.04

*p* values correspond to one-way ANOVA. Different letters indicate significant differences according to Tukey’s post hoc test (*p* < 0.05).

## Data Availability

The data presented in this study are available on request from the corresponding authors.
